# Glucocerebroside-Containing Milk Concentrated Powder Suppresses Oxidative Stress and Photoaging in the Skin of Hairless Mice

**DOI:** 10.3390/antiox11091804

**Published:** 2022-09-13

**Authors:** Dakyung Kim, Eun-hye Choi, Ju Young Lee, Hye-Jeong See, Hye-Jin Kim, Yunhi Cho, Ok-Kyung Kim, Jeongmin Lee

**Affiliations:** 1 Department of Medical Nutrition, Kyung Hee University, Yongin 17104, Korea; 2 R&D Group, Maeil Health Nutrition Co., Ltd., Pyeongtaek 17714, Korea; 3 Research Institute of Clinical Nutrition, Kyung Hee University, Seoul 02447, Korea; 4 Division of Food and Nutrition and Human Ecology Research Institute, Chonnam National University, Gwangju 61186, Korea

**Keywords:** buttermilk, milk phospholipids, oxidative stress, skin photoaging, UVB

## Abstract

This study investigated the protective effects of glucocerebroside-containing buttermilk concentrated powder (GCBM) on oxidative stress and photoaging in ultraviolet B (UVB)-irradiated hairless mice. We measured antioxidant enzyme activities, collagen synthesis-related pathways, and moisturizing-related factors in the dorsal skin of mice. We observed that dietary supplementation with GCBM increased antioxidant enzyme activity and decreased pro-inflammatory cytokine expression in the UVB-irradiated dorsal skin. Furthermore, dietary supplementation with GCBM inhibited wrinkle formation by suppressing the JNK/c-FOS/c-Jun/MMP pathway and stimulating the TGF-βRI/Smad3/procollagen type I pathway. Dietary supplementation with GCBM also increased skin moisturization by stimulating hyaluronic acid and ceramide synthesis in the dorsal skin. Therefore, buttermilk powder supplementation helps prevent photoaging and can be used as an effective component in developing anti-photoaging products.

## 1. Introduction

As a vital organ, the skin defends the body’s internal structures from the external environment. Epidermis, the top layer of the skin, retains moisture and protects the body from external stimuli, while dermis, the middle layer, provides strength and elasticity to the skin by forming matrix components [1,2]. Cutaneous aging is caused by a complex biological phenomenon triggered by intrinsic (genetically determined physiological aging) and extrinsic factors (temperature, pollution, and ultraviolet (UV) irradiation). Premature skin aging is caused by UV irradiation and is called photoaging [3,4].

Maintaining skin moisture and elasticity is key to healthy skin. The stratum corneum of a healthy epidermis contains 15–20% moisture; below 10% moisture, the skin becomes dry and rough, which induces wrinkle formation. Structural components, including collagen, hyaluronic acid, and ceramides, help moisturize and strengthen the protective skin barrier [5]. UV irradiation induces the overproduction of reactive oxygen species (ROS) in the epidermis, leading to the secretion of pro-inflammatory cytokines, which are involved in the up-regulation of inflammatory reactions. UVB-mediated pro-inflammatory cytokines, such as IL-1β, IL-6, and TNF-α, inhibit the expression of type-1 procollagen via protein degradation in the extracellular matrix by activating matrix metalloproteinases (MMPs) [6,7]. Collagen, with over 90% existing as collagen type I, is the most abundant protein in the dermis and the main component of the matrix in the skin; thus, collagen degradation plays an important role in the formation of wrinkles. Furthermore, hyaluronic acid and ceramides in the skin play essential roles in regulating physiological water balance by preventing dryness. The loss of these substrates in the skin results in cutaneous aging [8,9].

In the butter-making process, the butter is churned from the cultured cream, and the remnant liquid is known as buttermilk [10]. Buttermilk contains more phospholipids than skim milk because it contains a milk fat globule membrane; phospholipids in buttermilk have potential cholesterol- and blood-pressure-lowering as well as anti-cancer effects [11,12]. Milk phospholipids have been shown to downregulate the expression of nuclear factor kappa-B (NF-κB) in UVB-irradiated hairless mice, demonstrating their skin-protective effect [13]. Due to the low amount of phospholipids contained in milk with an amphiphilic chemical structure, several studies have attempted to improve the separation methods of such compounds [14]. In this study, we investigated the effect of buttermilk powder on skin health, including oxidative stress, inflammation, wrinkle formation, and moisturization, in UVB-irradiated hairless mice. In addition, we determined whether buttermilk powder affected hyaluronic acid, sphingomyelin, and ceramide content in the skin.

## 2. Materials and Methods

### 2.1. Preparation of Glucocerebroside-Containing Milk Concentrated Powder

Glucocerebroside-containing milk concentrated powder (GCBM) was provided by Maeil Health Nutrition Co., Ltd. (Seoul, Korea). GCBM is developed from milk. Briefly, pasteurized cream separated from the milk was removed from anhydrous milk fat using centrifugation. It was filtered and concentrated to GCBM and stored in an air-tight container at −20 °C until use. It consists of phospholipids 13.0 ± 2.0%, glycosphingolipids 3.8 ± 1.1%, and glucocerebroside 0.22 ± 0.05%.

### 2.2. UVB Irradiation-Induced Photoaging in Mice

Five-week-old male SKH-1 hairless mice were purchased from SaeRonBio (Uiwang, Korea) and maintained in ventilated cages. All mice were randomly divided into the following groups (8 mice per group): normal control (NC), control (C; UVB irradiation), positive control (PC; UVB irradiation with dietary supplementation of *l*-ascorbic acid at 100 mg/kg body weight [b.w.]), GCBM-100 (UVB irradiation with dietary supplementation of GCBM at 100 mg/kg/b.w.), GCBM-200 (UVB irradiation with dietary supplementation of GCBM at 200 mg/kg/b.w.), and GCBM-500 (UVB irradiation with dietary supplementation of GCBM at 500 mg/kg/b.w.). Photoaging was induced by UVB, following the method described previously [15]. The dorsal skin of SKH-1 hairless mice was exposed to a UVB lamp (Sankyo Denki Co., Yokohama, Japan) thrice per week for 8 weeks to induce photoaging of the skin. The minimal erythema dose (MED) was set at 150 mJ/cm^2^. The UVB dose schedule comprised UVB irradiation at 1 MED (150 mJ/cm^2^) in week 1, 2 MED (300 mJ/cm^2^) in week 2, 3 MED (450 mJ/cm^2^) in week 3, and 4 MED (600 mJ/cm^2^) in weeks 4 to 8. At the end of eight weeks, the mice were sacrificed by orbital venipuncture. We measured the dorsal wrinkle area using Image J (version 1.8.0, NIH, Bethesda, MD, USA). This study was approved by the Institutional Animal Care and Use Committee of Kyung Hee University (KHGASP-20-702).

### 2.3. Histological Observation

Skin tissues from the mice were isolated and fixed in 10% buffered formalin. Fixed skin tissues were embedded in paraffin after dehydration. The paraffin blocks were sliced into 5 μm sections and stained with hematoxylin and eosin (H&E).

### 2.4. Antioxidant Enzyme Activity in Dorsal Skin

Skin tissues from mice were lysed, and the activities of superoxide dismutase (SOD), catalase, and glutathione peroxidase (GPx) were measured using the superoxide dismutase 1 (SOD1) (Mouse) ELISA Kit, catalase activity colorimetric/fluorometric assay kit, and glutathione peroxidase activity colorimetric assay kit (BioVision Inc., Milpitas, CA, USA), respectively.

### 2.5. Protein Extraction and Western Blot Analysis

Skin tissues were lysed using CelLytic MT cell lysis reagent (Sigma-Aldrich, St. Louis, MO, USA). Equal amounts of total protein (100 μg) from skin tissues were dissolved in 4X NuPAGE LDS sample buffer (Life Technologies, Gaithersburg, MD, USA) and were separated using SDS-PAGE and transferred onto a nitrocellulose membrane. Membrane blocking, incubation with primary and secondary antibodies, visualization, and analysis were performed following the methods described previously [15]. Primary antibodies (JNK, p-JNK, c-Fos, p-c-Fos, c-Jun, p-c-Jun, MMP-1, MMP-3, MMP-9, Smad3, p-Smad3, HAS2, CerS4, and β-actin; 1:1000), and secondary antibody (anti-rabbit IgG HRP-linked antibody; 1:5000) were purchased from Cell Signaling (Beverly, MA, USA).

### 2.6. Isolation of Total RNA and Real-Time Reverse Transcription Polymerase Chain Reaction (RT-PCR)

Total RNA from dorsal skin tissues was isolated using an RNeasy Mini Kit (Qiagen, Valencia, CA, USA). Complementary DNA synthesis was performed using an iScript cDNA Synthesis Kit (Bio-Rad, Hercules, CA, USA). PCR amplification was conducted for 40 cycles at 95 °C for 15 s, 58 °C for 15 s, and 72 °C for 30 s with SYBR Green PCR Master Mix (Bio-Rad) and primer pairs (Table 1). Data analysis was performed using the 7500 System SDS version 1.3.1 (Applied Biosystems, Foster City, CA, USA).

### 2.7. Skin Hydration

At the end of eight weeks, hydration of the dorsal skin was measured using moisture analyzer (Howskin, Seoul, Korea) under standardized conditions of 22–24 °C and 55–60% humidity.

### 2.8. Levels of Hyaluronic Acid and Sphingomyelin

The skin tissues from the mice were lysed, and hyaluronic acid and sphingomyelin levels were determined using the hyaluronic acid ELISA Kit (Biovision Inc., S. Milpitas Boulevard, Milpitas, CA, USA) and the Sphingomyelin Assay Kit (Abcam, Cambridge, UK), respectively, according to the manufacturers’ protocols.

### 2.9. Ceramide Content

The epidermis was isolated from dorsal skin tissues by incubation in Hank solution (Balanced Salt Solution) with DisapseⅡ (2.4 unit/mL, Roche, Germany) at 4 °C for 16 h. The isolated epidermis was homogenized using a Polytron homogenizer, and lipids were extracted using Folch solution (CHCl_3_:MeOH = 2:1, *v*/*v*). The supernatant was obtained by centrifugation, and the solution contained CHCl_3_:H_2_O = 1:1 (*v*/*v*) and H_2_O:CHCl_3_:MeOH = 0.8:1:1 (*v*/*v*/*v*). The obtained lower layer was dried with N_2_ gas, and CHCl_3_:MeOH = 1:1 (*v*/*v*) was added. Ceramide fraction was obtained using CHCl_3_:MeOH: acetic acid = 9.5:0.45:0.05 (*v*/*v*/*v*) up to 10 cm by high-performance thin-layer chromatography (HPTLC). The fractions were scanned using a TLC III scanner (CAMAG, Muttenz, Switzerland), and ceramide levels were expressed as μg/mg protein.

### 2.10. Statistical Analysis

All data are presented as mean ± standard deviation (SD). Differences among groups were evaluated using one-way analysis of variance (ANOVA) and Duncan’s multiple range tests implemented in SPSS for Windows (SPSS PASW Statistics 22.0, SPSS Inc., Chicago, IL, USA). Differences were considered statistically significant at *p* < 0.05.

## 3. Results

### 3.1. GCBM Attenuated Wrinkle Formation and Oxidative Stress in UVB Irradiation-Induced Photoaging

**Figure 1:**
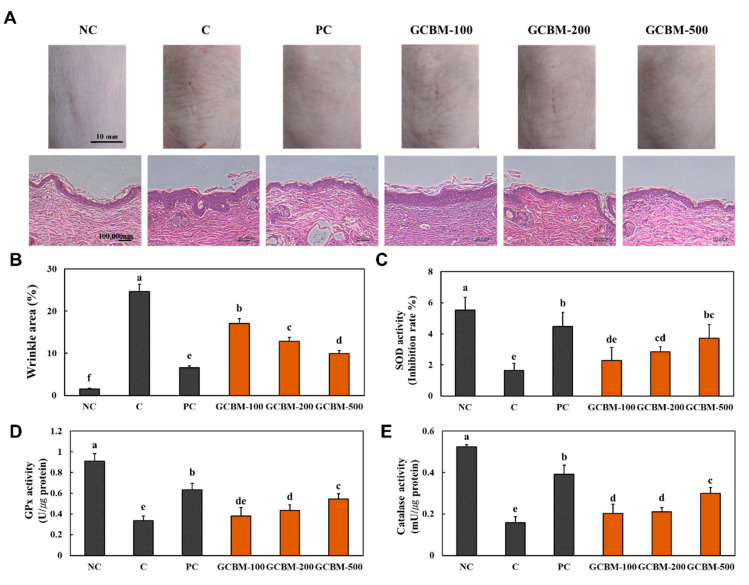
Effects of GCBM on morphological and histopathological changes (**A**), wrinkle area (**B**), and antioxidant activities of SOD (**C**), GPx (**D**), and catalase (**E**) in the dorsal skin of UVB irradiation-induced photoaging in mice. Normal control (NC), control (C; UVB-irradiation), positive control (PC; UVB-irradiation with diet containing *l*-ascorbic acid at 100 mg/kg/body weight [b.w.]), GCBM-100 (UVB-irradiation with dietary supplementation of GCBM at 100 mg/kg/b.w.), GCBM-200 (UVB-irradiation with dietary supplementation of GCBM at 200 mg/kg/b.w.), and GCBM-500 (UVB-irradiation with dietary supplementation of GCBM at 500 mg/kg/b.w.). Values are presented as mean ± SD. Different letters (a > b > c > d > e > f) indicate a significant difference, with *p* < 0.05, as determined with Duncan’s multiple range test.

We confirmed that there was no significant difference in weight gains, food efficiency ratio (FER), or organ weights among the groups (data not shown). The morphological and histopathological changes in the skin after UVB irradiation-induced photoaging are shown in Figure 1A. We found that the oral administration of *l*-ascorbic acid and GCBM attenuated the morphological and histopathological changes in the dorsal skin of mice induced by UVB irradiation. In addition, compared with the control group, the oral administration of *l*-ascorbic acid and GCBM significantly decreased the wrinkle area (Figure 1B). Figure 1C–E shows that the antioxidant enzyme activity of SOD, GPx, and catalase was lower in the UVB irradiation control group than in the NC group. Oral administration of *l*-ascorbic acid and GCBM increased the antioxidant enzyme activity compared to the control group (*p* < 0.05).

### 3.2. GCBM Suppressed Skin Inflammation in UVB Irradiation-Induced Photoaging

**Figure 2:**
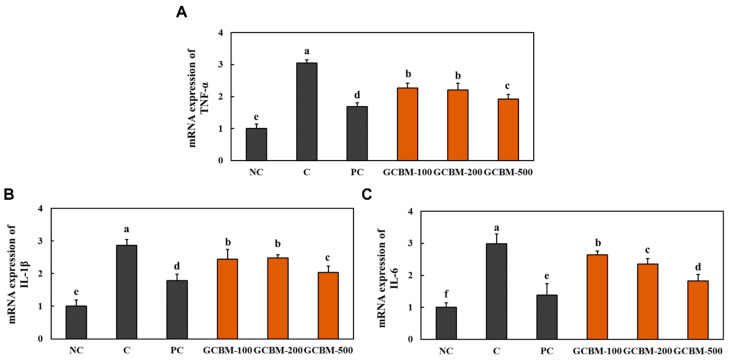
Effects of GCBM on the mRNA expression of *TNF-α* (**A**), *IL-1β* (**B**), and *IL-6* (**C**) in the dorsal skin of UVB irradiation-induced photoaging in mice. Normal control (NC), control (C; UVB-irradiation), positive control (PC; UVB-irradiation with diet containing *l*-ascorbic acid at 100 mg/kg/body weight [b.w.]), GCBM-100 (UVB-irradiation with dietary supplementation of GCBM at 100 mg/kg/b.w.), GCBM-200 (UVB-irradiation with dietary supplementation of GCBM at 200 mg/kg/b.w.), and GCBM-500 (UVB-irradiation with dietary supplementation of GCBM at 500 mg/kg/b.w.). Values are presented as mean ± SD. Different letters (a > b > c > d > e > f) indicate a significant difference, with *p* < 0.05, as determined with Duncan’s multiple range test.

The mRNA expression levels of *TNF-α*, *IL-1β*, and *IL-6* were significantly increased in the dorsal skin of mice that experienced UVB irradiation-induced photoaging compared to the NC mice. However, the mRNA expression levels of *TNF-α, IL-1β*, and *IL-6* in the exposed area were significantly decreased in PC and GCBM groups than in C (Figure 2) (*p* < 0.05).

### 3.3. GCBM Increased the Activation of Elasticity Factors in UVB Irradiation-Induced Photoaging

**Figure 3:**
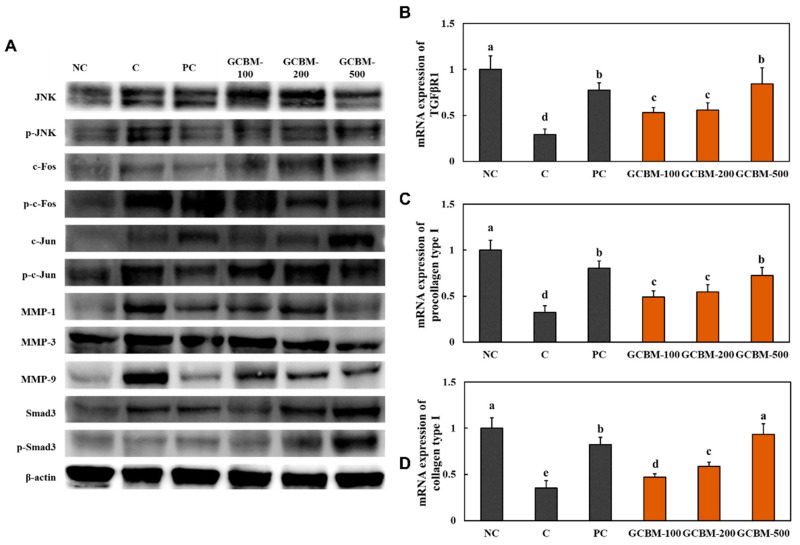
Effects of GCBM on protein expression of JNK/c-FOS/c-Jun/MMPs/Smad3 pathway (**A**), TGF-*β RI* mRNA (**B**), *procollagen Type I* mRNA (**C**), and *collagen Type I* mRNA (**D**) in the dorsal skin of UVB irradiation-induced photoaging in mice. Normal control (NC), control (C; UVB-irradiation), positive control (PC; UVB-irradiation with diet containing *l*-ascorbic acid at 100 mg/kg/body weight [b.w.]), GCBM-100 (UVB-irradiation with dietary supplementation of GCBM at 100 mg/kg/b.w.), GCBM-200 (UVB-irradiation with dietary supplementation of GCBM at 200 mg/kg/b.w.), and GCBM-500 (UVB-irradiation with dietary supplementation of GCBM at 500 mg/kg/b.w.). Values are presented as mean ± SD. Different letters (a > b > c > d > e) indicate a significant difference, with *p* < 0.05, as determined with Duncan’s multiple range test.

We measured the expression levels of proteins involved in the wrinkle formation pathway, c-Jun N-terminal kinase (JNK)/c-FOS/c-Jun/MMPs/Smad3 pathway, and mRNA expression levels of elasticity factors—transforming growth factor-β receptor I *(TGF-β RI),* procollagen type I, and collagen type I—in the dorsal skin of mice exposed to UVB irradiation-induced photoaging. UVB irradiation with oral administration of *l*-ascorbic acid and GCBM decreased the protein expression levels of factors involved in the wrinkle formation pathway compared to the levels in the UVB irradiation control group (Figure 3A and Figure S1). Moreover, the mRNA expression levels of *TGF-β RI,* procollagen type I, and collagen type I in the dorsal skin of UVB irradiation-induced mice were significantly higher than those in the UVB irradiation control group mice (Figure 3B,C) (*p* < 0.05).

### 3.4. GCBM Increased Moisturizing-Related Molecules in UVB Irradiation-Induced Photoaging

**Figure 4:**
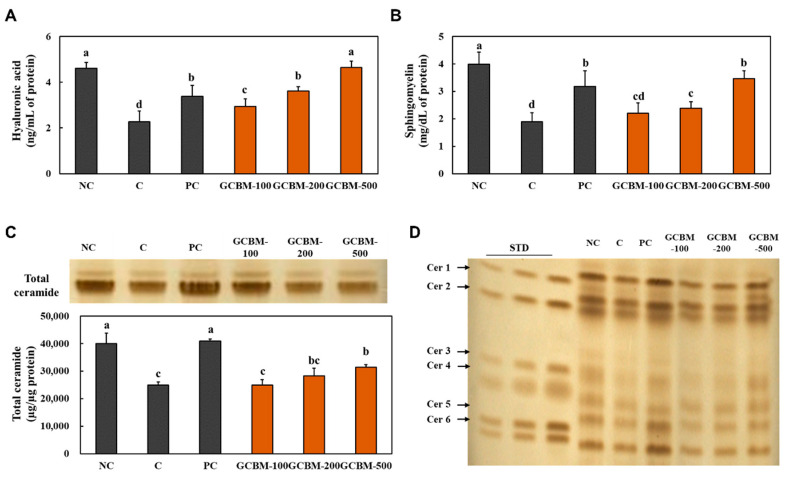
Effects of GCBM on the levels of hyaluronic acid (**A**), sphingomyelin (**B**), total ceramide (**C**), and the contents of ceramide 1~6 (**D**) in the dorsal skin of UVB irradiation-induced photoaging in mice. Normal control (NC), control (C; UVB-irradiation), positive control (PC; UVB-irradiation with diet containing *l*-ascorbic acid at 100 mg/kg/body weight [b.w.]), GCBM-100 (UVB-irradiation with dietary supplementation of GCBM at 100 mg/kg/b.w.), GCBM-200 (UVB-irradiation with dietary supplementation of GCBM at 200 mg/kg/b.w.), and GCBM-500 (UVB-irradiation with dietary supplementation of GCBM at 500 mg/kg/b.w.). Values are presented as mean ± SD. Different letters (a > b > c > d) indicate a significant difference, with *p* < 0.05, as determined with Duncan’s multiple range test.

We measured the levels of hyaluronic acid, sphingomyelin, and total ceramide, which play a role in skin moisturization in the dorsal skin of mice following UVB irradiation. The oral administration of *l*-ascorbic acid and GCBM showed a significant increase in the levels of hyaluronic acid, sphingomyelin, and total ceramide compared with that in the UVB irradiation control group (*p* < 0.05) (Figure 4A–C). In addition, we found that the oral administration of GCBM significantly increased the levels of ceramide 1, 2, 3, and 4 compared with those in the UVB irradiation control group (Figure 4D) (*p* < 0.05).

### 3.5. GCBM Increased Moisturizing Factors Activation in UVB Irradiation-Induced Photoaging

**Figure 5:**
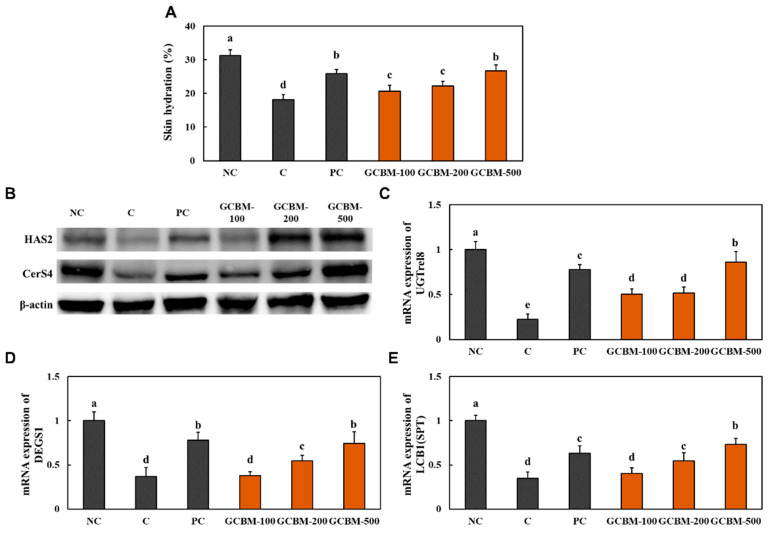
Effects of GCBM on skin hydration (**A**), protein expression of HAS2 and CerS4 (**B**), and mRNA expression of *UGTrel8* (**C**), *DEGS1* (**D**), and *LCB1* (**E**) in the dorsal skin of UVB irradiation-induced photoaging in mice. Normal control (NC), control (C; UVB-irradiation), positive control (PC; UVB-irradiation with diet containing *l*-ascorbic acid at 100 mg/kg/body weight [b.w.]), GCBM-100 (UVB-irradiation with dietary supplementation of GCBM at 100 mg/kg/b.w.), GCBM-200 (UVB-irradiation with dietary supplementation of GCBM at 200 mg/kg/b.w.), and GCBM-500 (UVB-irradiation with dietary supplementation of GCBM at 500 mg/kg/b.w.). Values are presented as mean ± SD. Different letters (a > b > c > d > e) indicate a significant difference, with *p* < 0.05, as determined with Duncan’s multiple range test.

Figure 5A shows that skin hydration was lower in the UVB irradiation control group than in the normal control group. However, UVB irradiation with oral administration of *l*-ascorbic acid and GCBM showed a significant increase in skin hydration compared to that in the UVB irradiation control group.

We measured hyaluronic acid synthesis-related factors, protein expression of hyaluronic acid synthase 2 (HAS2), mRNA expression of *UGTrel8* (UDP-glucuronic acid/UDP-*N*-acetylgalactosamine transporter; *SLC35D2*), ceramide synthesis-related factors, protein expression of ceramide synthase 4 (CerS4), mRNA expression of *delta 4-desaturase, sphingolipid 1* (*DEGS1*), and *serine palmitoyltransferase* (*LCB1*) in the dorsal skin. We determined that the oral administration of *l*-ascorbic acid and GCBM caused a significant increase in both hyaluronic acid synthesis-related factors and ceramide synthesis-related factors compared with those in the UVB irradiation control group (Figure 5B–E and Figure S1) (*p* < 0.05).

## 4. Discussion

This study investigated whether GCBM can protect the skin from UVB irradiation-mediated oxidative stress and photoaging. We showed that dietary supplementation with GCBM, especially at 500 mg/kg/b.w, attenuated the morphological and histopathological changes induced by UVB irradiation, including deep wrinkles and increased epidermal thickness. In addition, GCBM dietary supplementation suppressed UVB irradiation-induced oxidative stress and inflammation in the dorsal skin of mice.

By analyzing the relative reducing activity, sulfhydryl content, and ferrous and ferric iron-binding affinity, Wong et al. previously confirmed the antioxidant activity of buttermilk solids [16]. They found that buttermilk solids had strong reducing activity, and the affinity of scavenging ·OH activity by buttermilk solids contributed to both reducing and iron sequestering activities. Moreover, Conway et al. reported that heat-denatured buttermilk proteins and those hydrolyzed by pepsin and trypsin could scavenge free radicals [17]. Our findings and those of previous studies suggest that oral administration of buttermilk has antioxidant effects that defend against the decrease in antioxidant enzyme activity induced by UVB irradiation.

Collagen type I, the most abundant collagen in the skin, decreases due to UVB irradiation, resulting in reduced elasticity and the formation of deep wrinkles. Transforming growth factor-β receptor I (TGF-β RI) stimulates collagen type I production via small mothers against decapentaplegic homolog (Smad) phosphorylation. Collagen degradation occurs due to MMP-mediated proteolysis through the phosphorylation of JNK, c-Fos, and c-Jun. UVB irradiation inhibits the TGF-βRI/Smad3/procollagen type I pathway and stimulates the JNK/c-FOS/c-Jun/MMPs pathway, leading to collagen degradation and wrinkle formation [18,19]. We found that dietary supplementation with GCBM stimulated the TGF-βRI/Smad3/procollagen type I pathway and suppressed the JNK/c-FOS/c-Jun/MMPs pathway in the dorsal skin of mice exposed to UVB irradiation. These findings indicate that dietary supplementation with GCBM suppresses collagen degradation and stimulates collagen synthesis during UVB irradiation-induced wrinkle formation.

This study showed that GCBM dietary supplementation increased skin moisturizing factors, including hyaluronic acid, sphingomyelin, and total ceramide. Epidermal ceramides are the largest lipid component of lamellar sheets present in the intercellular space of the stratum corneum, play a dominant role in moisture retention, and function as a skin barrier. Ceramides are synthesized from glucocerebroside (also called glucosylceramide) using glucocerebrosidases [20,21]. Several studies have demonstrated that oral administration of glucocerebroside improves epidermal water loss and barrier function by forming permeability barriers. Dietary glucocerebrosides can be hydrolyzed to ceramide in mucosal cells after absorption [22,23]. Ueda et al. demonstrated that orally administered ceramide is gradually distributed in the dermis following absorption and is transferred from the dermis to the epidermis in rats [23].

Ceramides consist of a sphingoid base linked to an acyl chain, and the most abundant sphingoid base is sphingosine in mammals. Sugawara et al. investigated the cellular uptake of sphingoid bases after treatment with an inhibitor of P-glycoprotein that contributes to the selective absorption of sphingosine in Caco-2 cells. They found that the P-glycoprotein inhibitor increased the cellular concentrations of sphingoid bases from plants and yeast but did not affect sphingosine uptake. Thus, they suggested that sphingoid bases, except for sphingosine, are absorbed poorly from the digestive tract [24].

Since dietary supplementation with GCBM increased the total ceramide levels in the dorsal skin, we investigated whether the GCBM-induced increase in ceramide levels was caused by stimulation of the factors involved in ceramide synthesis. We showed that dietary supplementation with GCBM increased ceramide synthesis-related factors CerS4, *DEGS1,* and *LCB1* in the dorsal skin. These findings suggest that GCBM promotes ceramide synthesis in the skin, although GCBM contains glucocerebroside. Further studies on whether ceramides released from dietary glucocerebroside-containing GCBM can be delivered to the skin are needed to confirm the underlying molecular mechanism.

## 5. Conclusions

We confirmed that dietary supplementation with GCBM prevented UVB irradiation-induced oxidative stress and photoaging in the skin of hairless mice. In addition, GCBM treatment inhibited wrinkle formation by suppressing the JNK/c-FOS/c-Jun/MMP pathway and stimulating the TGF-βRI/Smad3/procollagen type I pathway. GCBM treatment also increased skin moisturization by stimulating hyaluronic acid and ceramide synthesis in the dorsal skin. Our results suggest that supplementation with buttermilk powder may help prevent skin photoaging by inhibiting collagen degradation and regulating skin moisturization.

## Figures and Tables

**Table 1:** Primer sets used for real-time RT-PCR.

Gene	Accession Number	Sequence
*TNF-α* (M)	NM_001278601.1	F: 5′-CAC CGT CAG CCG ATT TGC-3′
		R: 5′-TTG ACG GCA GAG AGG AGG TT-3′
*IL-1β* (M)	XM_006498795.5	F: 5′-GAT GAT AAC CTG CTG GTG TGT GA-3′
		R: 5′-GTT GTT CAT CTC GGA GCC TGT AG-3′
*IL-6* (M)	NM_001314054.1	F: 5′-CGC TAT GAA GTT CCT CTC TGC AA-3′
		R: 5′-CAC CAG CAT CAG TCC CAA GAA-3′
*TGF-β RI* (M)	AF271072.	F 5′-CAT CCT GAT GGC AAG AGC TAC A-3′
		R 5′-TAG TGG ATG CGG ACG TAA CCA-3′
*Procollagen type I* (M)	NM_008788.2	F 5′-TTA CGT GGC AAG TGA GGG TTT-3′
		R 5′-TGT CCA GAT GCA CTT CTT GTT TG-3′
*Collagen type I* (M)	NM_007742.4	F 5′-GAC CGT TCT ATT CCT CAG TGC AA-3′
		R 5′-CCC GGT GAC ACA CAA AGA CA-3′
*UGTrel8* (M)	XM_006517374.4	F: 5′-TTC CTC ATC GTG CTG GTC AA-3′
		R: 5′-TTG GTG AGG GAA AAC CGT ATG-3′
*DEGS1* (M)	NM_007853.5	F 5′-CCG GCG CAA GGA GAT CT-3′
		R 5′-TGT GGT CAG GTT TCA TCA AGG A-3′
*LCB1(SPT)* (M)	AF003823.1	F 5′-AGC GCC TGG CAA AGT TTA TG-3′
		R 5′-GTG GAG AAG CCG TAC GTG TAA AT-3′
*GAPDH* (M)	NM_001289726.1	F 5′-CCC CAC ACA CAT GCA CTT ACC-3′
		R 5′-TTG CCA AGT TGC CTG TCC TT-3′

## Data Availability

The data is contained within this article and supplementary file.

## Supplementary Materials

The following supporting information can be downloaded at: www.mdpi.com/article/10.3390/antiox11091804/s1, Figure S1. Quantification of the western blot bands’ relative expression in Figure 3 and Figure 5.
