# Supplementary material for: Glucocerebroside-Containing Milk Concentrated Powder Suppresses Oxidative Stress and Photoaging in the Skin of Hairless Mice
Source: Antioxidants (Basel). 2022 Sep 13;11(9):1804. doi: 10.3390/antiox11091804 (PMC9495600; doi:10.3390/antiox11091804)
Supplement: Supplementary file 1 [file antioxidants-11-01804-s001.zip › antioxidants-1871849-supplementary.pdf]

Quantifications of Fig. 3A bands

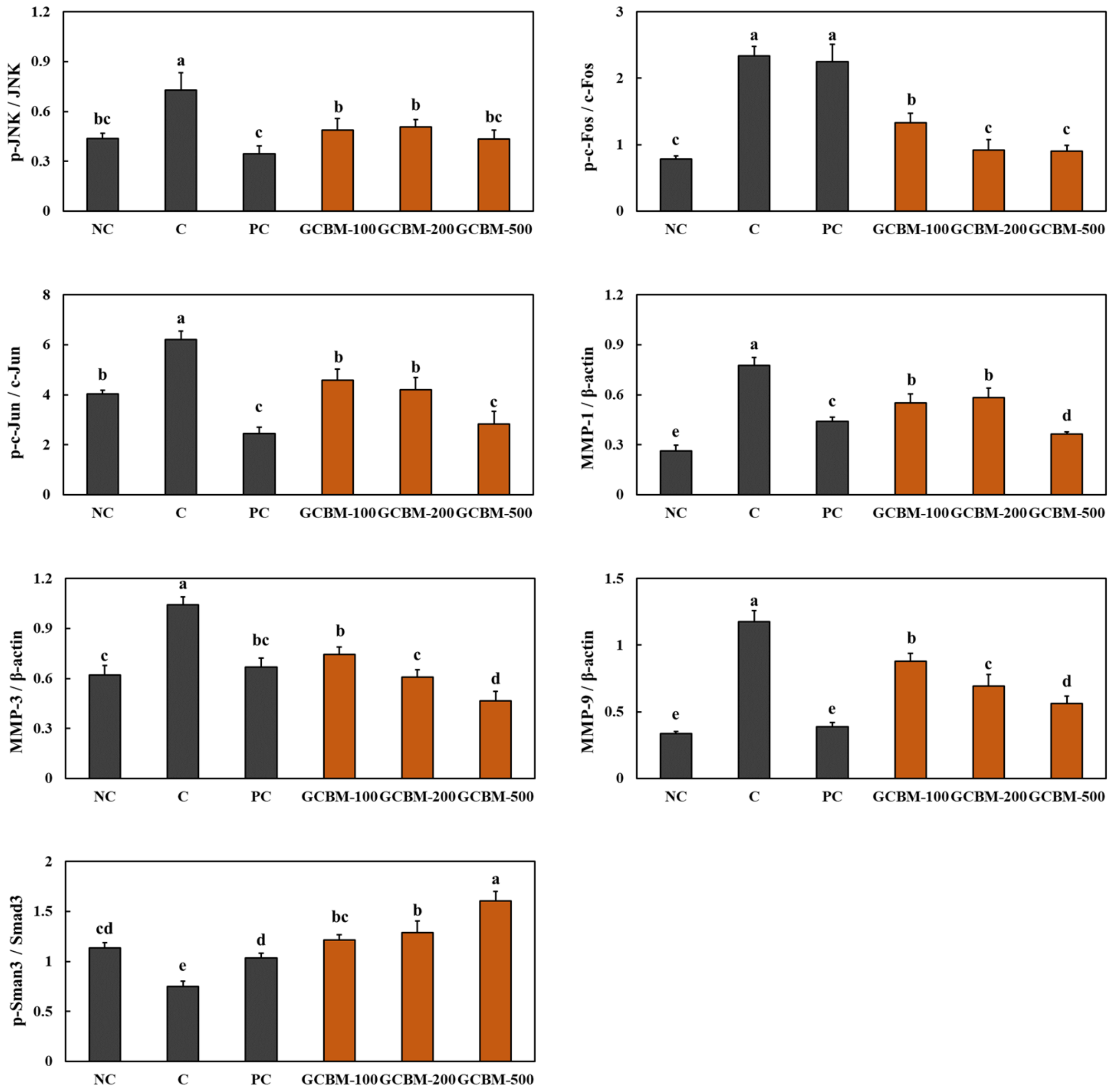

Quantifications of Fig. 5B bands

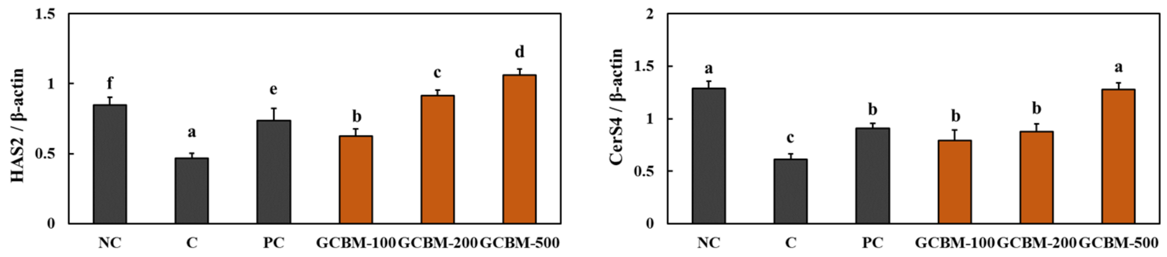

Figure S1. Quantification of the western blot bands relative expression in Figure 3 and Figure 5.
